# Improving Medical Student Psychiatric Inpatient Placement Experience Through Interprofessional Learning – A Medical Education Quality Improvement Project

**DOI:** 10.1192/bjo.2025.10392

**Published:** 2025-06-20

**Authors:** Dinushi Marambe, Olamide Oyelade, Mukund Vandekar, Joel Lawson, Richard Brown

**Affiliations:** St Martin’s Hospital, Canterbury, United Kingdom

## Abstract

**Aims:** Medical students are known to be apprehensive about psychiatric inpatient placements. Anecdotally, they assimilate less well than other health profession students during shorter placements, and nurses and occupational therapists (OTs) may not understand their role in supervising and engaging with medical students. We applied the Model for Improvement to deliver a quality improvement (QI) project to enhance student self-rated experience on placement by exploring induction, participation in a community of practice, engagement with the broader multidisciplinary team (MDT) and consider student safety.

**Methods:** The project involved clinical ward staff from two inpatient wards, the Medical Education team, the QI team, medical students from GKT School of Medical Education and resident doctors. We applied the Model for Improvement using routinely collected end of placement data as a change measure for student experience, with an additional Sense of Belonging Survey. Interventions drew on the themes of improving communication, preparation, early assimilation and participation with the work of the wider team. These included early communication with the ward manager and ward administrator; provision of a guide and structured timetable; day one arrival for nursing handover at 7.30 am; week 1 prioritisation of working alongside nurses, nursing students, nursing assistants, OTs, physiotherapists and in therapeutic groups; and support from Foundation doctors.

**Results:** Analysis of End of Placement Survey Data showed an early sustained trend of improvement, most marked in the themes of:

The block was well organised and the team was prepared for my arrival.

Teachers were welcoming and were expecting me for timetabled sessions.

During this placement I had opportunities to learn with the multidisciplinary team.

Students participated in the OT and physio therapeutic programme in a way that had never happened before and felt positive about the experience. Nursing colleagues felt strongly medical students should understand their role in inpatient care and welcomed their involvement. Students qualitatively reported feeling more welcome by other professions, felt safer and that they got to know patients more rapidly.

**Conclusion:** Medical students welcome changes to placement planning to enhance their engagement and assimilation with the mental health ward MDT. Ward MDT members place importance on medical students understanding their role and contribution. With planning, student placements can enhance interprofessional learning. We suspect that better student integration enhances their safety on wards. Foundation doctors can lead placement planning and embed change sustainably. Quality Improvement methodology can be applied to enhance medical education.

